# Progesterone as a Neuroprotective Agent in Neonatal Hypoxic-Ischaemic Encephalopathy: A Systematic Review

**DOI:** 10.1159/000521540

**Published:** 2022-11-25

**Authors:** Ming-Te Lee, Roisin McNicholas, Lawrence Miall, Nigel Simpson, Kevin C.W. Goss, Nicola J. Robertson, Paul Chumas

**Affiliations:** ^a^Senior House Officer, Department of Neurosurgery, Leeds General Infirmary, Leeds, UK; ^b^Medical Student, The Medical School, The University of Leeds, Leeds, UK; ^c^Consultant Neonatologist, Leeds Children's Hospital, Leeds, UK; ^d^Senior Clinical Lecturer, University of Leeds, Honorary Consultant Obstetrician and Gynaecologist, Leeds General Infirmary, Leeds, UK; ^e^Consultant Neonatologist, Princess Anne Hospital, University Hospital Southampton NHS Foundation Trust, Southampton, UK; ^f^Professor of Perinatal Neuroscience, University College London, Consultant Neonatologist, University College London Hospital NHS Trust, London, UK; ^g^Consultant Neurosurgeon, Leeds General Infirmary, Leeds, UK

**Keywords:** Progesterone, Neuroprotection, Hypoxia-ischemia, Hypoxic-ischemic encephalopathy, Neonatal brain injury, Perinatal brain injury, Brain injury

## Abstract

Hypoxic-ischaemic encephalopathy (HIE) in the newborn baby is a major contributor to neonatal mortality and morbidity across the world. Therapeutic hypothermia (TH) is the current standard treatment for moderate to severe HIE, but not all babies benefit. Potential neuroprotective actions of progesterone (PROG) include anti-apoptotic, anti-inflammatory, and anti-oxidative effects and reduction of energy depletion, tissue/cellular oedema, and excitotoxicity. In pre-clinical studies of neonatal HIE, PROG has neuroprotective properties but has not been the subject of systematic review. Here, our objective was to evaluate the evidence base for PROG as a potential therapeutic agent in HIE. The PICO framework was used to define the following inclusion criteria. Population: human neonates with HIE/animal models of HIE; intervention: PROG +/− other agents; comparison: V.S. control; outcome: pathological, neurobehavioural, and mechanistic outcome measures. Medline, EMBASE, and CINHAL were then searched between August to October 2018 using pre-defined medical subject heading and keywords. Study inclusion, data extraction, and risk of bias (ROB) analysis using the SYRCLE ROB tool were carried out by two authors. 14 studies were included in the review. They typically displayed a high ROB. This systematic review suggests that PROG reduced neuropathology and reduced neurobehavioural deficits post-hypoxic-ischaemic (HI) insult in 8 and 3 studies, respectively. However, there was sex dimorphism in the effects of PROG. In addition, there are limitations and biases in these studies, and there remains a need for well-designed large pre-clinical studies with greater methodological quality to further inform the efficacy, safety, dose, timing, and frequency of PROG administration. With such data, large animal studies could be planned combining PROG administration with and without TH.

## Introduction

Despite significant advancement in the field of neonatology, hypoxic-ischaemic encephalopathy (HIE) remains one of the major contributors to early neonatal mortality, along with prematurity, infections, and low birth weight [1]. Currently, HIE occurs in 1–3/1,000 live births in developed countries and up to 26/1,000 live births in low-resource settings [2]. It contributes significantly to neonatal mortality and morbidity, with long-term neurodevelopmental complications being seen in up to 25–60% of survivors [3]. The diagnosis of HIE is based on the presence of neurological dysfunction manifesting in the form of neonatal encephalopathy, the hallmarks of which include altered consciousness, commonly associated with respiratory depression, abnormality of muscle tone and power, abnormal cranial nerve function and seizures [4]. Neonates with suspected HIE are classified according to one of the staging classifications (e.g., modified Sarnat), which takes into consideration the level of consciousness, muscle tone, tendon reflexes, primitive reflexes, and autonomic function and classifies into stage I (mild), stage II (moderate), and stage III (severe) [5].

Therapeutic hypothermia (TH) with intensive care support is the routine treatment for neonates with moderate-to-severe HIE in high-resource settings. Clinical trials have shown that TH reduces mortality and morbidity rates of HIE [6], without increasing major disability in survivors. The benefits of TH on survival and neurodevelopment outweigh the short-term adverse effects which included cardiac events, such increase in episodes of sinus bradycardia, haematological sequela, such as thrombocytopenia and leukopenia, and endocrinological events, such as hypoglycaemia [6]. According to the UK National Institute of Clinical Excellence (NICE) guidance, TH is usually started within 6 h of birth once diagnosis of HIE is established, and the infant is cooled to a core target temperature of 33.5°C with the aim to ameliorate the evolution of brain injury following the initial hypoxic insult [7]. A more recent review by Nair and Kumar highlighted possible limitations of TH, in that its effect on severe HIE has not been fully established and that the implementation of TH is challenging in resource-poor settings [8].

Over the last two decades, TH adjunct therapies for HIE have been studied; xenon, erythropoietin, topiramate, levetiracetam, N-acetylcysteine, and melatonin have shown promise in pre-clinical models [9, 10, 11, 12, 13, 14, 15]. A search of ClinicalTrials.gov reveals ongoing human studies investigating the roles of hyperbaric oxygen, caffeine, erythropoietin, umbilical cord milking, autologous cord blood, xenon, melatonin, allopurinol, and sildenafil [16, 17, 18, 19, 20, 21, 22, 23, 24].

The neuroprotective effect of PROG has been studied in adult animal models of various neurological conditions including traumatic brain injury, neural ischaemia, spinal cord injury, peripheral nerve injury, demyelinating disease, neuromuscular disorders, and seizures [25]. Studies investigating PROG in animal models of ischaemic brain injury have shed light on biochemical mechanisms of PROGs neuroprotection properties. PROG reduces post-hypoxic-ischaemic cellular oedema by maintaining the structural integrity of the blood-brain barrier (BBB) [26, 27, 28]. PROG suppresses post-hypoxic-ischaemic cellular apoptosis via the PI3K/Akt/GSK-3β Pathway and triggers the release of brain-derived neurotrophic factor [29, 30, 31, 32]. PROG also reduces post-hypoxic-ischaemic cellular inflammation by reducing the expression of several immune mediators such as TNF-α, NF-κB, and IL-6, amongst others [27, 30, 33, 34, 35]. PROG contributes to the reduction in oxidative stress following injury [35]. Lastly, PROG has been shown to confer neuroprotection by contributing to synaptogenesis [36], maintaining cellular structure [37], and attenuating the post-ischemic injury NMDA-induced rise in intracellular calcium concentration [38]. Clinical trials in humans have included phase I-III studies in neuro-trauma and a pilot study in premature babies [39, 40, 41]. The widely reported positive effects of PROG on the neuro-pathological and functional outcomes of adult animal models of ischemic brain injury allude to a potential neuroprotective effect of PROG in ischemic brain injury in a neonatal setting. To our knowledge, the potential neuroprotective role of PROG in HIE has not yet been the subject of a systematic review.

## Methods

The PICO framework was used to identify studies which met the inclusion criteria as follows:

Population: Human neonates with HIE or the equivalent in animal models.

Intervention: Studies focused on PROG administration to protect against HIE or limit the damage of neonatal hypoxia-ischaemia.

Comparison: Studies that compared the effects PROG to a control group receiving vehicle/no treatment or other group receiving alternative modes of treatment in human subjects with neonatal HIE or animal models of neonatal HIE.

Outcome: Studies that examined pathological and neurobehavioural outcomes and mechanistic properties of PROG in neonatal HIE were included.

### Literature Search

Electronic databases were searched between August-October 2018. Databases searched included: Medline (19462018), CINHAL (1937–2018), Embase (1974–2018). The search was structured to combine the following medical subject heading and keywords: (“progesterone” OR “P4”) AND (“Hypoxic Ischaemic encaphalopath*” OR “Hypoxic Ischemic encephalopath*” OR “Brain Ischemia” OR “Hypoxia, Brain”) AND (“Infant” OR “Neonat*” OR “Newborn” OR “Newly Born”). Prior to this literature search, we also conducted a search of official registers of systematic reviews and clinical trials (PROSPERO, UK Clinical Trials Gateway, Clinical Trials.gov and COCHRANES Library) which showed no active or past systematic reviews or clinical trials investigating PROG in HIE. The reference list of all studies deemed relevant was manually searched for additional relevant studies.

### Eligibility Criteria

Original, controlled studies in humans and in animal models that investigated the efficacy and/or the mechanisms of PROG in neonatal HIE were included regardless of the date of publication. Only papers written in English were included. Reviews, letters, comments, and conference abstracts were excluded.

### Study Inclusion

Records identified during the search were screened by two reviewers (ML and RM), at abstract and full text stage. Inter-rater agreement at abstract stage and full text-stage was 100%. Data were then extracted on a predefined data extraction form by the primary author (ML).

### Data Extraction

The primary author (M.L.) extracted data on various aspects of the study, including study design, number of subjects used, randomization of animals, blinding, age, sex, and species of the animals used, model of ischaemia, and the duration of exposure to hypoxia. ML also extracted data on the intervention used and the outcomes measures of the different studies. These include: whether PROG was given before or after HI insult, whether PROG was given antenatally or postnatally, dosing regimen of the PROG, the route of PROG administration, whether a maintenance dose of PROG was used, plasma PROG level, time to measurement of outcomes, whether the study supported neuroprotective property of PROG and any reported side effects.

### Quality Assessment

We (M.L. and R.M.) assessed the susceptibility to bias of each publication using the SYRCLE risk of bias (ROB) tool [42].

## Results

### Study Selection

**Fig. 1:**
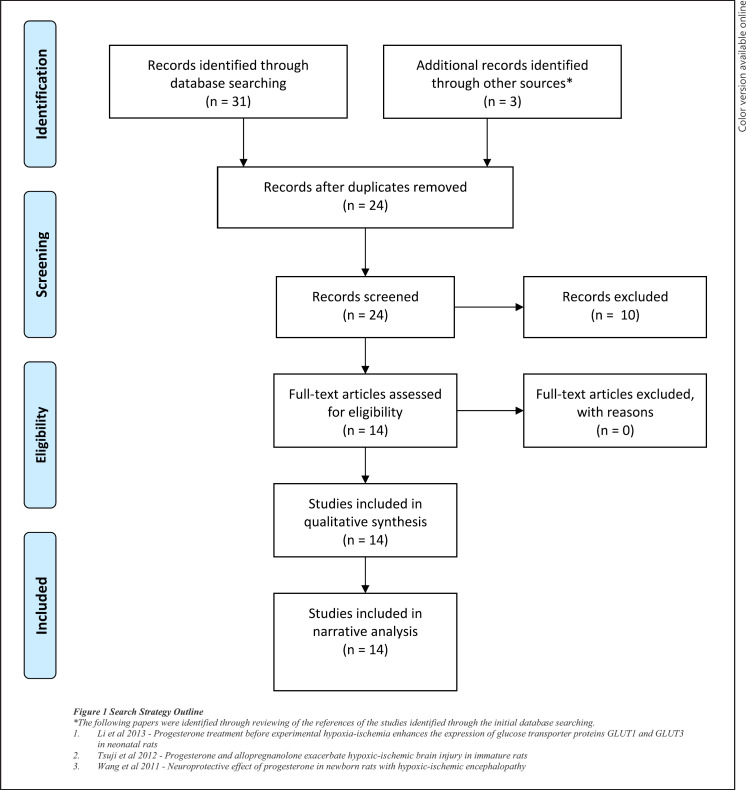
Search strategy outline.

The search identified 31 articles via electronic databases. Duplicates (10) were removed leaving 21 articles to be screened. After screening the titles and abstracts, 11 articles were included for full text screening. 3 studies were identified through reviewing the reference lists of the initial 11 included articles. 14 studies met the inclusion criteria (Fig. 1) and were included for the final review. No human studies were identified.

### Quality Assessment − ROB within Studies

**Fig. 2:**
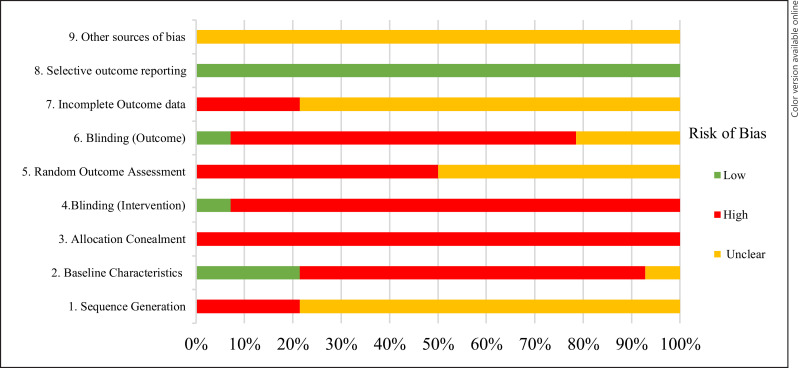
Risk of bias analysis. Bias assessed as per the SYRCLE tool for all 14 studies included.

ROB analysis was performed using the SYRCLE tool. The results of this assessment are outlined in Table 1 and summarized in Figure 2.

There is under reporting of details with regard to random sequence generation, allocation concealment, blinding of intervention, and outcome assessment for most studies. Between 21% and 100% of studies were judged to be at high ROB for a given criterion. No study reported details for all ten domains of the SYRCLE tool. Most studies exhibit unclear or high ROB.

No studies explicitly detailed the randomization of subjects into various experimental groups, despite the fact that 10 studies reported randomization of subjects. We defined age, sex, and weight of the animals as the three fundamental baseline characteristics. Only 3 studies (21.4%) adequately controlled for these three baseline characteristics. Only one study involved blinding of the intervention [47]. Only one study adopted blinding with regard to outcome assessment for all of the stated outcome measures in their studies [44]. Most studies had unclear or high risk for incomplete outcome data. Most studies did not provide adequate information to account for all the animal subjects in their studies. No studies performed concealment of allocation or provided explicit details on the performance of random outcome assessment. All 14 studies had low risk of selective outcome reporting. There is unclear risk of other sources of biases in all 14 studies.

Random housing of the animals is deemed not applicable to the studies that we have analysed and therefore is not included in the ROB analysis.

### Experimental Design Characteristic, Methodology, and Interventional Parameters

The key characteristics with regard to study design and methodology are summarized in Table 2 and Table 3 and further stratified into two cohorts based on whether PROG was given pre- or post-insult (refer to Table 4). 11 studies included experiments with pre-insult PROG, while 5 studies included experiments with post-insult PROG. 2 studies [43, 44] included experiments in which PROG was administered both before and after the HI-Insult, and for the purpose of this review, we have classified this as pre-insult administration of PROG. Fabres et al. [43] and Tskitishvili et al. [48] included experiments with pre- or post-insult administration of PROG; therefore, they contributed a count to both groups.

In the pre-treatment group, only Tsuji et al. [44]described blinding during measurement of outcome. There was no blinding of intervention in any of the experiments. Seven studies did not specify the sex of the animals used in their experiments. Three studies involved both female and male animal subjects in their experiments. One study [43] used only male rats in their experiments. Ten studies involved experiments that investigated the effect of PROG only on 7-day-old rats/mice. Tsuji et al. [44]also examined outcomes in 14- and 21-day-old rats, neither age would generally be considered in keeping with the human neonatal period [45]. It has been suggested that the brain in 7- to 10-day-old rats is equivalent to that of a human brain at term [46]. All the studies bar one (Li et al. [56] − right carotid artery ligation) utilized left carotid artery ligation as the model of ischaemia. All studies adopted postnatal administration of PROG via the intraperitoneal (IP) route.

In the post-treatment group, only Peterson et al. [47]described blinding of intervention and outcome measurement [47]. One study examined the effect of PROG combined with estradiol [48], while the rest examined that of PROG alone. Three studies recruited subjects of both sexes. One study utilized only male rats. One study did not specify the sex of the animals [48]. Four studies utilized 7-day-old rats/mice. One involved rats born around gestational day 22. Four studies utilized carotid artery ligation as the model of ischaemia. One study utilized transient bilateral uterine artery clamping in the pregnant rats on gestational day 18 as the method of inducing ischaemia [49]. All but one administered PROG via the IP route. Kawarai et al. did so via the subcutaneous route to the pregnant female animals.

All studies apart from Kawarai et al. [49] used a loading dose PROG that ranged from 1.6 to 16 mg/kg, the details of which can be found in Table 3. Tskitishvili et al. [48]administered either 1.6 or 16 mg/kg of PROG together with various doses of Estradiol (E2) and/or Estetrol (E4) in the relevant experiments in their study. Kawarai et al. [49] administered 0.1 or 0.01 mg/day of loading dose PROG to pregnant Wistar rats. They reported in their paper that 0.1 mg/day of PROG corresponds to 20.0 and 5.0 mg/kg/day on postnatal day (PD) 1 and PD 9, respectively [49].

In the pre-treatment group, 3 studies involved administration of further PROG doses after the loading dose. No studies measured plasma PROG level post PROG administration. In the post-treatment group, 4 studies involved administration of further PROG doses.

### Outcome Parameters − Mechanistic Outcomes

The various mechanistic properties of PROG as neuroprotectant in neonatal HIE are summarized in Table 5. Wang et al. obtained results that suggest PROG could restore normal Na^+^ and K^+^ exchange such that membrane ionic balance is maintained and cellular oedema is reduced [50]. Wang et al. [51] and Li et al. [52] found that pre-insult administration of PROG decreases the brain tissue content of Evans Blue Dye and Aquaporin-4 (AQP-4) at 24 h post-insult and up to 72 h post-insult [52]. Evans blue dye was used as a marker of BBB permeability in their studies. There is also a reduced expression of matrix metalloproteinase 9 (MMP9) in the pre-treatment group [51]. MMP is a type of proteinase that degrades extracellular matrices and damages the BBB thereby contributing to cerebral oedema and brain damage [51]. AQP-4 is a type of membrane protein found extensively in brain tissues that have been found to correlate with the formation of cerebral oedema in brain damage [53].

Glycogen synthase kinase-3β (GSK-3β) is a regulator of apoptosis in vivo and is also involved in the regulation of cell growth and development [50]. Activated protein kinase B (p-Akt) inhibits GSK-3β resulting in the maintenance of cell survival and inhibition of apoptosis [54]. Pre-insult treatment with PROG was found to increase the expression of p-Akt [54, 55] and to reduce the expression of GSK-3β in subjects' brain tissue [50, 55]. Conversely, Fabres et al. did not find such an effect at 48 h post HI insult in their experiments [43]. B-cell lymphoma 2 (BCl-2) is another regulator protein of cellular apoptosis, the downregulation of which results in cell apoptosis [32]. Pre-insult administration of PROG led to an increase in BCl-2 level in subjects' brain tissues [54].

Pre-insult PROG administration was also found to increase the expression of superoxide dismutase (SOD) and glutathione peroxidase [50]. Both are antioxidant enzymes that scavenge and remove toxic-free radicals, thereby reducing the oxidative stress that cells are exposed to [50]. Li et al. (2013) found that pre-insult administration of PROG resulted in an increased expression of glucose transporters 1 and 3 (Glut 1 and Glut 3) [56].

Multiple studies have assessed the anti-inflammatory property of PROG by examining the expression profile of inflammatory mediators and molecules. Two studies found that pre-treatment or post-HI insult treatment with PROG results in down regulation of the TNF signalling pathway. Specifically, they found a reduction in the expression of TNF-α or TNFR1/TRAF-6 when compared to the model group [57, 58]. The result found by Dong et al. [54, 57] was only in male mice. Two other studies found a reduction in NF-κB level in the PROG treatment group compared to the control group. One study also found a reduction in the expression of COX-2 and IL-1β in the PROG treatment group compared to the control group [59].

Lastly, pre-insult administration of PROG has been found to reduce the number of glial-fibrillary acidic protein (GFAP) positive cells [50]. GFAP is an astrocyte marker and has been implicated in the development of the inhibitory features of prolonged astrogliosis. It is also used as a marker of reactive astrocytes that are responding to central nervous system injuries [60].

### Outcome Measures − Pathological Outcomes

The pathological outcome measures are displayed in Table 6. Ten studies examined “pathological” outcomes to understand the neuroprotective effect of PROG [43, 44, 47, 48, 49, 50, 54, 55, 57, 58].

In the pre-treatment group, Wang et al. [50] found that neuronal apoptosis at 6h post-insult was significantly lower than that of the control group (*p* < 0.05). Li et al. [55] obtained similar finding at 24h post-HI insult. Conversely, Tsuji et al. [44] found that pre-insult administration PROG correlated with the reduction of ipsilateral brain hemispheric volume (treatment group: 271 ± 11 mm^3^ vs. control: 345 ± 14 mm^3^, *p* < 0.001) and also exacerbated brain insult in all four brain regions evaluated (i.e., the cortex, striatum, hippocampus and thalamus) in 7-day-old rats [44]. Fabres et al. [43] found that the volume of infarction after PROG administration at all time points was not reduced when compared to the control group (*p* > 0.05). Tskitishvili et al. [48] found significantly higher number of intact cells in the hippocampal dentate gyrus tissue in the group pre-treated with E4 alone or in combination with PROG and/or E2, when compared to control. In the same study, MAP-2-positive (marker for grey matter) area ratio was found to be significantly higher in groups treated by E4 alone or in combination with PROG, when compared control [48]. PROG was found to reduce HI-insult induced neuronal cellular damage − specifically the extent of cellular cavitation, when compared to control groups [54, 57].

In the post-treatment group, all five studies examined “pathological” outcome measures. In contrast to the results obtained by Fabres et al. [43], Peterson et al. [47] found that PROG significantly reduced whole-brain tissue loss at 7-weeks post-HI insult in males only. They also found that PROG significantly reduced tissue insult in three out of six brain tissue sections in males but not in the control or female groups. Similarly, Dong et al. found that post-insult PROG administration reduced the infarction area in male mice compared to control group, but this was not observed in female mice [58]. In contrast to the results obtained by Tsuji et al. [44] Kawarai et al. [49] found greater number of neurons in the cortex and hippocampal CA1, and also more oligodendrocytes in the corpus callosum in the PROG-treated group compared to control. Similar results were obtained by Tskitishvili et al. [48] as mentioned previously.

### Outcome Measures − Neurobehavioural Measures

Only three studies included “neurobehavioural” outcome measure**s** to assess the effect of PROG on the functional outcomes of the animals studied, as displayed in Table 7. All three studies involved post-insult administration of PROG. Peterson et al. [47] found that neonatal pups (Sprague-Dawley) with HI insult treated with PROG, when assessed on a number of functional outcome measures consistently had smaller deficits than that of the sham-operated and/or vehicle-treated groups, albeit these differences are mostly nonsignificant. Specifically, they found that when analysed as a trend over time, the latency to fall off time in the Rotarod test was significantly less in the male PROG-treated group compared to the sham-group and vehicle-treated group [47]. In contrast, Kawarai et al. [49] found that treatment with PROG restored the latency-to-fall off of the rats to the level of the sham-group. Dong et al. [58] found that PROG treatment in male mice subjected to HI insult, significantly improved the impairment of cognitive, spatial, and memory abilities as seen through the Morris Water Maze test, compared to untreated male mice. This was not observed in female mice.

## Discussion

PROG is a naturally occurring progestin produced primarily by the ovary but also within the central nervous system [61]. It is implicated in pregnancy maintenance but also modulation of neuronal excitability. PROG levels increase continuously during pregnancy, with the level in the third trimester 7-fold that of the first trimester, and 22-fold that seen in the non-pregnant luteal phase [62, 63, 64, 65]. Previous studies have reported maternal PROG concentrations to range from 70 nmol/L in the first trimester to over 500 nmol/L by the late third trimester [66, 67, 68]. It has also been found that PROG level in maternal serum drops sharply post-parturition, by about 16-fold by post-partum days 2–3 [64, 69, 70, 71]. In foetuses, PROG plasma levels reach as high as 400–600 ng/mL [49]. The highest concentration was observed at gestational age 33 and 36 weeks with a decline near the end of the pregnancy, with the concentrations in the mother and the newborn infant decreasing to non-pregnancy levels within 24 h of delivery [72]. Previous studies have not identified a clear sex difference in cord and postnatal PROG levels [71, 73]. Given that the foetal brain is exposed to high levels of PROG, it has been hypothesized that PROG plays a key role in the protection of immature neurons.

The pathophysiology of neonatal HIE presents multiple potential therapeutic targets at various time points throughout the course of the disease [74]. Following hypoxia-ischaemia, the encephalopathy evolves over time and there are three key phases. The first “acute” phase (primary phase) is triggered by hypoxia, ischaemia and cellular energy depletion, which leads to anaerobic metabolism, glutamate excitotoxicity, and calcium influx. Calcium influx then results in the production of free radicals, activation of a range of pro-apoptotic enzymes, lipid peroxidation, culminating in cell death [4, 8, 74, 75]. Following hypoxia-ischaemia, there is a transient recovery of the energy depletion on resuscitation of the baby − this period is often described as the therapeutic window [4, 8] when early interventions can reduce subsequent brain damage [76]. Much of our understanding of cerebral metabolism following HI has evolved through magnetic resonance spectroscopy through which we have shown that latent phase duration is inversely related to insult severity. Thereafter, typically 6–24 h after a moderate-to-severe primary insult, there is a secondary decrease in high energy phosphates which parallels the development of irreversible cellular injury. This secondary phase is marked by the onset of seizures [4, 8, 74], secondary cytotoxic oedema, accumulation of cytokines, and mitochondrial failure. Mitochondrial failure is a key step leading to delayed cell death. The degree of energy failure influences the type of neuronal death during early and delayed stages [77]. There is evidence that active pathological processes occur for weeks, months, and years after a hypoxic-ischaemic insult; this has been termed tertiary brain injury [4, 8, 78].

**Fig. 3:**
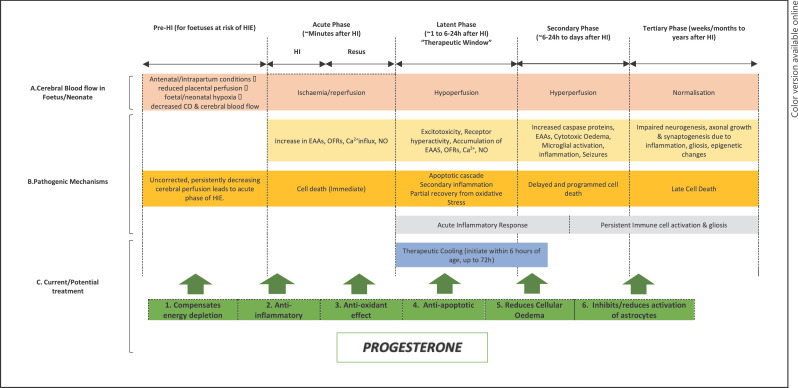
Schematic diagram (adapted from Hassell et al. [75], Nair et al. [8] and Douglas-Escobar and Weiss [4]), illustrating the different pathological phases of cerebral injury after onset of hypoxia-ischaemia. Progesterone may be useful as an adjuvant to therapeutic cooling by exerting neuroprotective effects during the primary (acute) phase, latent phase and secondary phase of the disease process of HIE. Most animal studies adopted pre-insult administration of PROG due to convenience of such study design. We appreciate the difficulty in pre-emptive administration of PROG to at risk foetuses but propose that PROG may be useful when given both before and after HI injury. The proposed mechanisms of neuroprotection by PROG are listed in no particular chronological order relative to the pathological phases of HIE. CO, cardiac output, HI, hypoxia-ischaemia, EAAs, excitatory amino acids, NO, nitric oxide, OFRs, oxygen free radicals.

The potential neuroprotective effects of PROG in neonatal HIE can be categorized into anti-apoptotic, anti-inflammatory, anti-oxidative, compensation of energy depletion, reduction of tissue/cellular oedema, and reduction of excitotoxicity via inhibition of astrocytes. These effects of PROG as a potential neuroprotectant in HIE are summarized in Figure 3 (in no chronological order).

This systematic review confirms that, overall, PROG is neuroprotective in neonatal HIE − with only two studies showing no positive effects of PROG on animals with HI injury. Of these, Fabres et al. concluded that PROG did not show positive or negative effects in newborn animals subjected to neonatal HI [43]. Tsuji et al. concluded that PROG exacerbated brain injury in PD7 and PD14 rats [44]. It should be noted that D7-D10 in rats is equivalent to that of a human brain at term [46]; we suggest the results from D14 rats is of less relevance to neonatal HI. However, the finding that PROG or allopregnanolone (Allo) given before and after HI insult in D7 rat pups was detrimental (more volume loss and increased pathology damage scores) compared to that in controls is clearly of potential concern. The authors propose that this finding might be due to the reverse effect (excitatory rather than inhibitory) of GABA receptors in neonates [44]. The authors note that GABAergic currents become inhibitory at different ages in different species and in humans occur in the last trimester of pregnancy [79]; they suggest this potential detrimental effect of PROG may not apply in the human setting. This study is also at variance with the other studies in this review. It is also worth noting that Peterson et al. [47] who concluded PROG to be protective, did observe a trend to a decrease in latency to fall off time in the rotarod test in males; however, they attributed this to a dose-response effect rather than a true detrimental effect of PROG itself.

### Sex Differences in the Effects of PROG

We identified potential sex-related differences in the effect of PROG in HIE. Dong et al. [58]and Peterson et al. [47]described statistically significant neuroprotective effect of PROG only in male animals. Male sex has been identified a predisposing factor for HIE in a retrospective population-based cohort study by Wu et al. [80]. On a pathophysiological level, sex dimorphism post-HI insult has also been identified in cell death pathway, oxidative stress, and microglial activation [81, 82]. This observation could also be due to the fact that PROG receptor (PR) expression is relatively higher in male neonatal male pups within specific brain regions while there is essentially no PR expression in female pups of similar age [83]. Previous studies investigating sex difference in various outcomes in rat models of HI demonstrated that male rats showed greater brain volume loss, behavioural deficits, and cell death compared to females [84]. Another study found sex-dependent differences in brain MRI findings in rats, which suggests possible neuroanatomical differences between the two sexes at the same age points [85]. Taking the above into account, it could be that since male rodents have a greater pathophysiological activity, suffer greater damage, and display more deficits than female rodents of the same age, the corresponding therapeutic effect conferred by PROG would be proportionately more pronounced in males. These data emphasize the need for future studies to be stratified by sex.

Such differences have also been observed in human studies in other neurological conditions. Ment et al. reanalysed their data from the “Indomethacin IVH prevention trial” in very low birth weight infants and found that indomethacin was associated with higher verbal scores at 3–8 years in males only [86]. Donder et al. [87] found that boys who sustained traumatic brain injury had statistically significantly lower neuropsychological performance compared to girls at 1-year follow-up. In another study, Lauterbach et al. [88] identified a female advantage in cognitive recovery from respiratory distress syndrome (RDS). Despite previously demonstrated gender differences in animal models of neonatal HIE subjected to hypothermia in terms of behavioural and pathological outcomes, no relevant human data are available [89, 90]. Surprisingly, various follow-up studies of hypothermia in human neonates with HIE did not stratify their results according to sex [91, 92, 93, 94].

### Could PROG Be Given to Human Neonates Safely?

In view of the lack of human studies involving the replacement of PROG in neonates with HIE, we have reviewed the literature looking for any studies where PROG was administered either to the mother or to neonates in order to see if there is any evidence of safety in this setting. In the OPPTIMUM trial of vaginal PROG for prevention of preterm birth in high risk women, PROG had no significant effect on 2-year neurodevelopmental outcomes [95]. On the other hand, an earlier systematic review by Sotiriadis et al. found that PROG given to women decreased the risk for neonatal mortality and common morbidity (RDS, NICU admission, and composite adverse outcomes) in singleton pregnancies [96]. More recently, Romerto et al. [97] have found that PROG given to women with twin gestation and short cervix was associated with a significant reduction in neonatal death, RDS and composite neonatal morbidity and mortality when compared to controls.

A preterm infant is prematurely extracted from a PROG-rich environment at an earlier developmental stage compared to term infants and may subsequently miss out on any protective effects that PROG might have [98]. Previous studies have indicated that 13–27% of premature infants with perinatal acidosis subsequently develop HI brain injury [99, 100]. Trotter et al. [101] conducted a small pilot study involving replacement of PROG and E2 to female preterm neonates. At follow-up at 15 months of age, no significant difference was found in the Bayleys Scales of Psychomotor and Mental between the hormone-treated group and the control group. However, the average psychomotor development index for the hormone-treated group was found to be within the normal range, as opposed to that of the control group [102], suggesting a potential positive effect. They also investigated the effect of replacement of PROG and E2 in preterm infants on bronchopulmonary dysplasia [103] and identified no difference in the motor and cognitive attainments between the treatment and control groups [104]. More importantly, no adverse effects of steroid replacement on short- or long-term outcomes were detected.

A subsequent Cochrane review found no significant beneficial effect of replacement of E2 and PROG in premature neonates less than 30 weeks' gestation [105]. However, these authors advised that a properly powered randomized controlled trial is required to establish the efficacy and safety profile of administering PROG and/or E2 to preterm infants [105].

It is worth noting another study involving randomized administration of an analogue of allopregnanolone (Ganaxolone), to preterm guinea pig pups by Shaw et al. [98]. They found that postnatal administration of ganaxolone improved neurobehavioural outcomes and also increased myelination of the hippocampus and subcortical white matter in preterm pups as compared to controls. This was generally consistent with the findings from our systematic review, given that allopregnanolone is a neuroactive metabolite of PROG. However, they also observed that preterm pups administered ganaxolone had poorer weight gain, showed greater level of sedation and higher mortality rates compared to controls [98]. The increased mortality rates were attributed to acute (<24 h) respiratory events and hypotonia secondary to allopregnanolone, and suggestion was made for further studies to investigate optimal dose that best balances neurological benefits and minimal side effects [98].

### If PROG Were to Be Given for Human Neonatal HI, When Should It Be Administered, at What Dose, via What Route, and for How Long?

Our systematic review suggests the ideal PROG loading doses that are associated with improved outcome after HI insult to be between 8 and 16 mg/kg in rat/mice models of HIE. In the studies [89, 92, 93] on premature neonates noted above, far higher doses (22.5 mg/kg/day) were used with the dose tailored to mirror PROG plasma levels in the range known for normal human foetus in utero, i.e., 300–600 ng/mL of PROG [101].

All the animal studies involved postnatal administration of the PROG doses through the IP route apart from Kawarai et al. [49] which adopted subcutaneous administration of PROG to pregnant rats. In the human neonatal study by Trotter et al. PROG was administered through the intravenous route and subsequently via the transdermal route at about 6-folds higher with no adverse effects [101]. The transdermal route, while easy to use, does not ensure achievement of therapeutic plasma level of PROG [106], and in human neonates, intravenous administration is likely the most feasible route.

It is also important to determine the ideal timing for the administration of PROG in relation to the HI insult. In this review, eleven studies involved experiments that entailed pre-insult administration of PROG. However, from a clinical perspective, post-HI administration is far more relevant. Of the five studies with experiments that adopted post-HI insult administration of PROG, only one study found no benefits of PROG [43]. Two studies found that PROG is neuroprotective only in male animals [47, 58]. Nevertheless, future dedicated large-scale animal studies are needed to establish the optimal dosing and duration of treatment, although intuitively it would make sense to cover the latent and secondary phases of HI, i.e., >6–48 h post-insult, and possibly up to 1 week, in order to utilize the neuroprotective mechanistic properties of PROG against specific pathogenic processes of HIE at the corresponding time windows.

### Limitations of Current Studies

Most of the studies included in this systematic review involved experiments entailing pre-HI insult administration of PROG. Whilst this is of less clinical relevance than postnatal “rescue” treatment, we included these studies in our review as they still provide useful information on the potential mechanistic properties and side-effects of PROG. Unfortunately, this review draws attention to frequent design flaws in the animal studies − particularly in relation to randomization, specifying the sex of the animals used and blinding of investigators at both time of administration of agents and that of assessment outcomes [44, 47, 49, 58]. Frustratingly, such limitations seem to be widespread amongst animal research [31, 96]. The lack of standardization of age, temperature, length of time in hypoxia, and outcome measures used makes it difficult to compare results, not only between the various groups receiving PROG but perhaps more importantly with other potential therapeutic agents for neonatal HIE.

Only two studies accounted for the body temperature of the animal subjects in their experiments. Tsuji et al. [44] reported no difference in rectal temperatures between the experimental groups at any time points before or up to 7 h after HI insult but no data was provided in their paper and no elaboration was done to specify which particular experimental groups were being referred to. Tskitishvili et al. [48] reported that higher doses of PROG (16 mg/kg/day) in various combinations with E4 with/without E2 was associated with significantly lower rectal temperature of the animal subjects, compared to the sham-group or the other groups with lower dose PROG (1.6 mg/kg/day). It has been noted that small reduction in brain temperature in neonatal animals is associated with a reduction of the pathogenic processes of HI brain injury [107] while an increase in brain temperature of neonatal animal models of HI resulted in greater magnitude of brain injury and behavioral deficits [108, 109]. Laptook et al. proposed that the association between hyperthermia and death or disability could be due to three reasons, that brain injury raises body temperature, that hyperthermia results in brain injury, or that hyperthermia is in itself an indicator of underlying pathogenic process [110]. It is therefore essential that future relevant animal studies further explore the relationship between body/brain temperature and PROG administration, to shed more light on whether PROG can be a realistic adjuvant therapy to TH.

Whilst trying to study another rare paediatric condition (brain tumours), the Gilbertson Group in the University of Cambridge have recently reported the use of an MDT approach to their animal studies comprising statisticians, biologists, chemists, pharmacologists, and clinicians [111]. “The pMDT met weekly to design, conduct and review preclinical studies that closely recapitulate multimodality clinical trials.” We would argue that the study of neonatal HIE could potentially benefit enormously from such a robust approach − which might allow multiple potential agents to be assessed and the best then taken forward to clinical trial.

Lastly, at the time of preparing the manuscript for submission (October 2021), a repeat literature search identified a recent paper published by Fabres et al. in 2020 [112] which reaffirmed the previous findings that PROG does have neuroprotective properties on the brains of neonatal animal models of HIE. In contrast to their study in 2018 in which they measured the various outcomes at 48 h post-HI insult, Fabres et al. [112] in their more recent 2020 study measured the outcomes at a more delayed time point of 7 days post-HI insult. They found results which further support the neuroprotective effects of PROG. However, as this is the only new paper identified since this systematic review was first conducted in 2018, and to maintain the integrity and validity of the initial search and vetting process and risk of bias assessment, this paper was not formally appraised in this systematic review.

## Conclusion

HIE remains a significant challenge in neonatal medicine and the search continues for agents to augment HT protection. PROG is a potential agent, and this review outlines the potential mechanisms of action of PROG and suggests that PROG is neuroprotective in most neonatal HIE animal studies; however, there may be important sex differences. Furthermore, PROG has been shown to be safe in neonates as may be explained by the inhibitory GABAergic currents found in human neonates. This review highlights limitations of the present animal studies and suggests the need for larger scale studies in small animals with greater methodological quality to further inform the efficacy, safety, dose, timing, and frequency of PROG administration. In particular, future small animal studies may be conducted using guinea pigs, which may have benefits for translational studies [113]. Once rodent data are more robust and show clear, promising protection then studies in large animals (e.g., piglet, sheep) can be undertaken to investigate whether PROG might augment TH in neonatal HIE.

## Statement of Ethics

The paper is exempt from Ethical Committee approval as no human or animal subjects are required for this systematic review.

## Author Contribution

M.-T.L. (corresponding author), R.M., L.M., N.G., K.C.W.G., N.J.R., and P.C. all made substantial contribution to the design, drafting/critical revision, and final approval of the work, and all agree to be accountable for all aspects of the work in ensuring that questions related to the accuracy and integrity of any part of the work are appropriately investigated and resolved. P.C. also contributed to the conception of the work.

## Conflict of Interest Statement

The authors have no conflict of interest to declare.

## Funding Sources

There was no formal funding for this work.

## Data Availability Statement

All data generated or analysed during this study are included in this article.

Further enquiries can be directed to the corresponding author.

## Figures and Tables

**Table 1:** Summary of the result of the assessment of the included studies using the SYRCLE risk of bias tool

Author/Year	Sequence generation	Baseline characteristics controlled?	Allocation concealment	Blinding (intervention)	Random outcome assessment	Blinding (outcome assessment)	Incomplete outcome data	Selective outcome reporting	Other sources of bias
Wang et al., 2011 [50]	?	− (gender not specified)	−	−	?	−	?	+	?

Tsuji et al., 2012 [44]	?	+			?	+ (for both neuropathological score and hemispheric volume measurement)	?	+	?

Li et al., 2013 [56]	?	− (number of each gender not specified)	−	−	?	−	?	+	?

Wang et al., 2013 [51]	?	− (gender not specified)	−	−	?	−	?	+	?

Li et al., 2014 [55]	?	− (no specific number of male or female rats given)	−	−	?	−	−	+	?

Li et al., 2014 [57]	?	− (gender not specified)	−	−			− (only 3 rats from reach of the 3 groups were involved in the analysis)	+	?

Wang et al., 2014 [59]	?	− (gender not specified)	−	−	?	−	?	+	?

Li et al., 2015 [54]	?	− (gender not specified)	−	−	?	−	? (only 3 rats from reach of the 3 groups were involved in the analysis)	+	?

Li et al., 2015 [52]	?	− (authors stated that they included an “unlimited” number of male and female rats)						+	?

Peterson et al., 2015 [47]	?	− (specific number of animals of each gender not specified, weight of animals not specified)		+		? (only stated that endogenous PROG measurement was done by independent laboratory)	+		?

Tskitishvili et al., 2016 [48]	−	− (no relevant information given)	−	−	−	−	?	+	?

Kawarai et al., 2018 [49]		?	−	−	−	? (only stated that cell counting was performed by a blinded investigator)	?	+	?

Fabres et al., 2018 [43]	−	+ (all males)	−	−	−	−	?	+	?

Dong et al., 2018 [58]	?	+	−	−	−	? (only for counting of FJ-B stained cells)	?	+	?

(+) indicates low risk of bias; (−) indicates high risk of bias; (?) indicates unclear risk of bias.

**Table 2:** Design and methods of the included studies

Author/Year	Study design	Random allocation	Blinding	Species	Sex	Age of animals	Model of ischaemia	Duration of ischaemia	Duration of exposure to hypoxia, h
Wang et al., 2011 [50]	Prospective, unblinded trial	Yes	No	Wistar rats	Not specified	Newborn	Left carotid artery ligation	Permanent	2.5

Tsuji et al., 2012 [44]	Prospective, single-blinded trial	Yes	Blinding during measurement of outcome	Wistar rats	Both	7D, 14D, 21D	Left carotid artery ligation	Permanent	2 (P7 rats), 1.33 (P14 rats), 0.83 (P21 rats)

Li et al., 2013 [56]	Prospective, unblinded trial	Yes	No	Sprague-Dawley rats	Both	7D	Right-carotid artery ligation	Permanent	2

Wang et al., 2013 [51]	Prospective, unblinded trial	Yes	No	Wistar rats	Not specified	7D	Left carotid artery ligation	Permanent	2.5

Li et al., 2014 [55]	Prospective, unblinded trial	Yes	No	Wistar rats	Both	7D	Left carotid artery ligation	Permanent	2.5

Li et al., 2014 [57]	Prospective, unblinded trial	Yes	No	Wistar rats	Not specified	7D	Left carotid artery ligation	Permanent	2.5

Wang et al., 2014 [59]	Prospective, unblinded trial	Yes	No	Wistar rats	Not specified	7D	Left carotid artery ligation	Permanent	2.5

Li et al., 2015 [54]	Prospective, unblinded trial	Yes	No	Wistar rats	Not specified	7D	Left carotid artery ligation	Permanent	2.5

Li et al., 2015 [52]	Prospective, unblinded trial	Yes	No	Wistar rats	Not specified	7D	Left carotid artery ligation	Permanent	2.5

Peterson et al., 2015 [47]	Prospective, single-blinded trial	Yes	Blinding to the interventions Endogenous PROG measurement done by independent laboratory	Sprague-Dawley rats	Both	7D	Right-carotid artery ligation	Permanent	3.5

Tskitishvili et al., 2016 [48]	Prospective, unblinded trial	Not Specified	not specified	Sprague-Dawley rats	Not specified	7D	Left carotid artery ligation + cutting	permanent	0.91

Kawarai et al., 2018 [49]	Prospective, partially single-blinded trial	Yes	No	Wistar rats	Both	Pups born around gestational day 22	Bilateral uterine artery clamping	Transient	NA

Fabres et al., 2018 [43]	Prospective, unblinded trial	Not Specified	No	Wistar rats	Male	7D	Left carotid artery ligation	permanent	1.5

Dong et al., 2018 [58]	Prospective, partially single-blinded trial	Yes	No	C57BL/6J mice	Both	7D	Left carotid artery ligation	permanent	1.25

**Table 3:** Intervention parameters of the relevant experiments and outcome measures of the included studies

Author/Year	PROG before/after HI insult?	PROG antenatally/postnatally?	PROG loading dose (mg/kg)	Route	PROG maintenance	Plasma PROG level post PROG administration	Time to outcome measurement	Outcome measure	Conclusion – PROG neuroprotective in NHIE?	Side-effects	Journal if
Wang et al., 2011 [50]	Before	Postnatal	4, 8, 16 (3 different dose regimes)	IP	None	Not measured	Rats that received 8 mg/kg PROG: 6, 24, 48, 72 h and 7 days	Mechanistic pathological	Yes	No mention	0.37 (2015)

Tsuji et al., 2012 [44]	Before + After	Postnatal	10	IP	6h, 24h post hypoxia at 10 mg/kg	Not measured	7D after Hypoxia	Pathological	No	Exacerbation of brain injury	4.483 (2017)

Li et al., 2013 [56]	Before	Postnatal	8	IP	None	Not measured	24 h after Hypoxia	Mechanistic	Yes	No mention	3.155 (2017)

Wang et al., 2013 [51]	Before	Postnatal	8	IP	None	Not measured	24 h after hypoxia	Mechanistic	Yes	No mention	1.848 (2017)

Li et al., 2014 [55]	Before	Postnatal	8	IP	None	Not measured	24 h after hypoxia	Mechanistic pathological	Yes	No mention	1.41 (2017

Li et al., 2014 [57]	Before	Postnatal	8	IP	None	Not measured	24 h after hypoxia	Mechanistic pathological	Yes	No mention	1.41 (2017)

Wang et al., 2014 [59]	Before	Postnatal	8	IP	None	Not measured	24 h after hypoxia	Mechanistic	Yes	No mention	1.848 (2017)

Li et al., 2015 [54]	Before	Postnatal	8	IP	None	Not measured	6 h, 24 h, 72 h after hypoxia	Mechanistic	Yes	No mention	0.833 (2017)

Li et al., 2015 [52]	Before	Postnatal	8	IP	None	Not measured	24 h after hypoxia	Mechanistic pathological	Yes	No mention	0.833 (2017)

Peterson et al., 2015 [47]	After	Postnatal	8	IP	2h, 24h, and 2, 3, 4, 5, 6 and 7D post hypoxia	Not measured	Up to 7 weeks post hypoxia	Pathological functional/neurobehavioural	Yes (in males)	7 pups died (no statistical significance)	3.173 (2017)

Tskitishvili et al., 2016 [48]	Before/After	Postnatal	1.6 or 16 per day, n combination with E2 and/or E4	IP	Pretreatment group: at days 2, 1 and day 0 (before HI insult)	Not measured	7D after hypoxia	Pathological	Yes (in combination with E4 with/without E2)	No mention	5.168 (2016)

Kawarai et al., 2018 [49]	After	Postnatal	0.1 or 0.01 mg/day*	SC	Postnatal D1 to D9	Not measured	PD 0 to PD 50	Pathological neurobehavioural	Yes	No mention	3.961

Fabres et al., 2018 [43]	Before/After/Before + After	Postnatal	10	IP	After group: 24h post HI Before + After group: 6 and 24h post HI	Not measured	Pathological and mechanistic measures (48h after hypoxia)	Mechanistic pathological	No	No mention	2.441

Dong et al., 2018 [58]	After	Postnatal	16	IP	6, 24, 48, 72, 96, 120h post HI insult at 16 mg/kg 144 and 168h post HI insult at 8 mg/kg and 4 mg/kg, respectively	Not measured	Western Blotting (24 h after HI) Fluoro-jade B staining (72 h after HI) Morris Water Maze (49 days after HI) MRI brain (8 weeks after HI)	Mechanistic pathological neurobehavioural	Yes (in males)	No mention	4.483 (2017)

* Higher dose of 0.1 mg/day roughly corresponds to 20.0 and 5.0 mg/kg/day on PD1 and PD9, respectively.

**Table 4:** Stratification of study methodology and intervention parameters according to whether PROG was given pre- or post-HI insult

Parameters	Pre-insult PROG (*n* = 11^a^)	Post-insult PROG (*n* = 5^a^)
Sex	7 NS, 3 both, 1 male only	1 NS, 3 both, 1 male only

Species	9 WR, 2 SDR	2 WR, 2 SDR, 1 × C57BL/6J

Age	Mostly 7D	Mostly 7D, Kawarai et al. [49] – pups born on gestational day 22

Ischaemia model	Mostly left carotid artery ligation	All but 1 adopted carotid artery ligation. Kawarai et al. [49] – B/L uterine artery clamping of mums at gestational day 18

Timing of PROG	All postnatally	All postnatally

Route	IP route	Kawarai et al. [49] used SC route, otherwise all IP

Dose	1.6–16 mg/kg	1.6–16 mg/kg^b^

Maintenance	Given in 3 experiments	Given in 4 experiments

Plasma PROG level^c^	Not measured	Not measured

SC, subcutaneous.

a Fabres et al. [43] and Tskitishvili et al. [48] included experiments with pre- or post-insult administration of PROG; therefore, they contributed a count to both groups. The total number of studies = 14.

b Kawarai et al. [49] administered 0.1 mg/day or 0.01 mg/day of PROG to newborn pups from PD1 to PD9. 0.1 mg/day/animal roughly correspond to 20.0 and 5.0 mg/kg/day on PD1 and PD9, respectively.

c None of the studies measured plasma PROG level in animals that have received PROG pre- or post-HI insult.

**Table 5:** Stratification of mechanistic properties of PROG as a neuroprotective agent according to whether PROG was given pre- or post-HI insult

Mechanistic property	Pre-insult PROG	Post-insult PROG
Compensates energy depletion	Li et al. [56] (↑ Glut 1, ↑ Glut 3)	

Anti-inflammatory	Li et al. [57] (↓ TNF-a, ↓ NF-κB)	Dong et al. [58] (jTRAF-6 and in J TRIF in males only)
	
	Wang et al. [50] (↓ COX-2, ↓ IL-1B)	
		
	Li et al. [54] (↓ NF-κB)	

Anti-oxidant effect	Wang et al. [50] (↑ SOD, ↑ GPX, ↓ MDA)	

Anti-apoptotic	Wang et al. [50] (↓ GSK-3B)	
		
	Li et al. [55] (↑ p-Akt, ↓ GSK-3B)	
		
	Li et al. [54] (↑ p-Akt, ↑ BCl-2)	
	
	Fabres et al. [43]^a^ (no effect on p-Akt)	Fabres et al. [43] (no effect on p-Akt)

Reduces tissue/cellular oedema	Wang et al. [50] (↓ cellular H_2_O, Na^2+^, Ca^2+^, NO, J K^+^)	
	
	Wang et al. [51] (↓ AQP-4, ↓ MMP-9, ↓ BBB permeability, j cerebral water content)	
	
	Li et al. [52] (↓ cerebral water content, ↓ AQP-4, ↓ BBB permeability)	

Excitotoxicity (reduces activation of astrocytes)	Wang et al. [50] (↓ GFAP)	

Barrier: GFAP, glial fibrillary acidic protein; TRAF-6, TNF receptor associated factor 6; TRIF, TIR-domain-containing adapter-inducting interferon-β. Glut, glucose transporters; TNF-α, tumour necrosis factor alpha; NF-κB, nuclear factor kappa-light chain-enhancer of activated B cells; COX-2, cyclooxygenase 2; IL-1B, interleukin-1β; SOD, superoxide dismutase; GPX, glutathione peroxidase; MDA, malondialdehyde; GSK-3B, glycogen synthase kinase 3β; p-Akt, phosphorylated protein kinase B; BCl-2, B-cell lymphoma 2 protein; AQP-4, Aquaporin-4; MMP-9, matrix metallopeptidase 9, BBB, blood-brain barrier.

a Fabres et al. [43] included experiments with pre- or post-insult PROG; therefore, it contributed a count to both groups.

**Table 6:** Stratification of pathological outcome measures of the various experiments according to whether PROG was given pre- or post-HI insult

Pre-insult PROG (*n* = 7)		Post-insult PROG (*n* = 5)	
Wang et al. [50]	↓ neuronal apoptosis	Peterson et al. [47]	↓ brain whole-brain tissue loss at 7 weeks post-HI insult in males only

Li et al. [55]	↓ neuronal apoptosis	Dong et al. [58]	↓ infarction area post-HI insult in males only

Li et al. [57]	↓ HI insult-induced cellular damage (SEM)	Kawarai et al. [49]	↑ neurons versus control
	
Tsuji et al. [44]	↑ brain injury in various regions ↓ Ipsilateral brain volume		
		
	No effect on rectal temperature		

Fabres et al. [43]^a^	No effect on volume of infarction	Fabres et al. [43]	No effect on volume of infarction

Tskitishvili et al. [48]^b^	↓ rectal temp ↑ no. of intact cells in hippocampus tissue ↑ grey matter ratio	Tskitishvili et al. [48]	↓ rectal temp ↑ no. of intact cells in hippocampus tissue ↑ grey matter ratio

Li et al. [54]	↓ HI insult-induced cellular damage (SEM)		

a Fabres et al. [43] included experiments with pre- or post-HI insult PROG; therefore, it contributed a count to both groups.

b Tskitishvili et al. [48] included experiments with pre- or post-HI insult PROG; therefore, it contributed a count to both groups.

**Table 7:** Stratification of neurobehavioural outcome measures of the various experiments according to whether PROG was given pre- or post-HI insult

Pre-insult PROG (*n* = 0)	Post-insult PROG (*n* = 3)	
None reported on neurodevelopmental outcomes	Peterson et al. [47]	Rotarod test: ↓ latency to fall off as a trend over time in males only
		
		Open field test: ↓ distance travelled in males, ↑ total time spent resting in males
		
		Sticky test: ↓ time taken to recognize sticky tab in males only
		
		Morris water maze: no difference between male groups
	
	Dong et al. [58]	Morris water maze: ↓ in latency to find the platform in males only
	
	Kawarai et al. [49]	Rotarod test: Restore latency to fall off time to normal
